# The efficacy and practicality of the Neurotrack Cognitive Battery assessment for utilization in clinical settings for the identification of cognitive decline in an older Japanese population

**DOI:** 10.3389/fnagi.2023.1206481

**Published:** 2023-08-31

**Authors:** Jordan M. Glenn, Kelsey Bryk, Jennifer R. Myers, John Anderson, Kaori Onguchi, Jacob McFarlane, Satoshi Ozaki

**Affiliations:** ^1^Neurotrack Technologies, Inc., Redwood City, CA, United States; ^2^Ebina General Hospital, Ebina, Japan

**Keywords:** cognitive decline, Alzheimer's disease, dementia, cognitive assessment, Neurotrack

## Abstract

**Introduction:**

Japan has the largest aging population with 33% of the population over the age of 60 years. The number of Japanese adults with dementia is estimated to be approximately 4.6 million, comprising nearly 15% of the older adult population. It is critical to administer cognitive assessments early in the disease state that have high reliability and low user burden to detect negative cognitive changes as early as possible; however, current preclinical AD detection methods are invasive, time-consuming, and expensive. A number of traditional and digital cognitive assessments are also available, but many of these tests are time-consuming, taxing to the user, and not widely scalable. The purpose of this study was to incorporate a digital cognitive assessment battery into a standard clinical assessment performed within a Japanese-based neuropsychology clinic to assess the diagnostic accuracy and the relationship between the digital Neurotrack Cognitive Assessment Battery (N-CAB) to traditional cognitive assessments.

**Methods:**

Healthy individuals and probable Alzheimer's patients completed the N-CAB, as well as two traditional cognitive assessments, the Mini Mental Status Exam (MMSE) and the Revised Hasegawa's Dementia Scale (HDS-R).

**Results:**

Our results demonstrate the Image Pairs hand-response phase of the N-CAB had the highest diagnostic accuracy with 95% sensitivity and 89% specificity to probable Alzheimer's disease. This was closely followed by the Symbol Match assessment, with a 96% sensitivity and 74% specificity to probable Alzheimer's disease. Additionally, Symbol Match and Path Points used in combination resulted in a sensitivity of 94%, specificity of 90%; a model with all N-CAB assessments resulted in a sensitivity and specificity of 100%. All N-CAB assessments had moderate to strong and significant correlations with the MMSE and HDS-R.

**Discussion:**

Together, this suggests that the N-CAB assessment battery may be an appropriate alternative for the clinical screening of cognition for earlier detection of Alzheimer's disease.

## Introduction

Characterized by a decline in mental ability severe enough to interfere with daily life, Alzheimer's disease (AD) poses a serious worldwide challenge as it relates to patients, their caregivers, and healthcare systems. Projections indicate that the global prevalence of AD is expected to triple to over 150 million individuals between 2015 and 2050 (Alzheimer's Disease International, 2018). In the United States alone, AD costs are projected to grow from USD$ 290 billion in 2019 to USD$ 1.1 trillion in 2050, representing a 400% increase, while AD diagnoses are projected to increase by approximately 150%, from 5.5 million to 13.8 million over that same timespan (Alzheimer's Association, 2018).

Among adults over 65 years, approximately one in five currently suffer from AD (Alzheimer's Association, 2015). This number has increased dramatically in the United States since 2000, with an increase of 145% in diagnoses (Alzheimer's Association, 2015). While these numbers are alarming, they are dwarfed by the massive growth in Japan's aging population. Compared with the rest of the world, Japan has the largest aging population with 33% of the population ≥ 60 years of age (Department of Economic Social Affairs: Population Division, 2017; United Nations, 2019). Furthermore, Japan's older population is projected to continue growing, reaching an unprecedented 42% of the population by 2050. This is critically important as the number of Japanese adults with dementia is estimated to be ~4.6 million, comprising nearly 15% of the older adult population (Okamoto, 2019). When individuals with mild cognitive impairment are included, this number rises to ~8.6 million, constituting 30% of Japanese older adults (Okamoto, 2019). The estimated cost of dementia in Japan in 2014, defined as the sum of costs for healthcare, formal care, and informal care, was approximately JPY¥ 14.5 trillion, or an estimated 3% of the nation's GDP (Sado et al., 2018).

Although 82% of adults over the age of 65 agree that testing their memory is important, only 16% receive regular cognitive assessments (Alzheimer's Association, 2019). This lack of early screening limits effective treatment and intervention strategies. Thus, it is critical to administer cognitive assessments early in the disease state which have high reliability and low user burden to detect negative cognitive changes as early as possible. Current preclinical AD detection methods include neuroimaging and biomarker assessment, such as amyloid-β and tau proteins (Tan et al., 2014; Ho et al., 2018), but these assessments are invasive, time-consuming, and expensive (Alzheimer's Association, 2019; Koo and Vizer, 2019). A number of traditional and digital cognitive assessments are also available, but many of these tests are time-consuming, taxing to the user, and not widely scalable (Lagun et al., 2011; Bott et al., 2018; Wadsworth et al., 2018). One such traditional cognitive screening test is the Mini-Mental State Examination (MMSE), which is one of the most commonly used cognitive assessments in Japan and internationally, but can take up to 15 min to administer, requires heavy administrator involvement, and has been copyrighted recently; therefore, there is a cost to use it (Folstein et al., 1975; Mitchell et al., 2011; Abe et al., 2021; Karimi et al., 2022). The Revised Hasegawa's Dementia Scale (HDS-R) is also a popular cognitive assessment for older adults in Japan that reports a cutoff score of 20 out of 30; however, it has been reported that this cutoff score may not be appropriate for all education levels; specifically, individuals with no formal education tend to score below the cutoff score, therefore making it difficult for the healthcare provider to determine whether the patient has a cognitive impairment (The Revised Hasegawa's Dementia Scale (HDS-R), 1994; Kounnavong et al., 2022). Both the MMSE and the HDS-R require verbal interaction between the test administrator and the patient, which can introduce many problems when assessing cognitive function. These include nervousness experienced by patients when answering questions in a healthcare setting and experiencing difficulty hearing or understanding the questions (Saji et al., 2019). In recent years, digitized cognitive assessments have become more readily available as technology advances and have demonstrated good validity when compared to their traditional paper-based cognitive assessments (Björngrim et al., 2019; Arioli et al., 2022). Digitized test batteries allow patients to self-administer cognitive assessments using technology that they may already be familiar with, which helps to remove the heavy involvement of the healthcare provider from the cognitive assessment while maintaining sensitivity to cognitive decline in older patients.

In order to be fit for purpose, measures of cognitive function must be reliable, sensitive, and valid to show meaningful change over time (Myers et al., 2022); however, they must also be short, simple to administer, fit within clinical workflows, and be adaptable to the nuances of the current healthcare system. As such, the purpose of this study was to incorporate the Neurotrack Cognitive Assessment Battery (N-CAB) into a standard clinical assessment performed within a Japanese-based neuropsychology clinic to (1) assess the ability of the N-CAB assessments and their composites to differentiate between healthy and AD subjects and (2) investigate the relationship between the N-CAB and traditional cognitive tests.

## Methods

### Study overview

This investigation utilized a real-world evidence (RWE) design where all participants were patients at a well-established neuropsychology clinic in Tokyo, Japan. Standard cognitive tests [the Mini-Mental State Examination (MMSE) and the Revised Hasegawa's Dementia Scale (HDS-R)] were administered by trained nurses and medical assistants in the clinic; these individuals also collected family history information. For patients who opted into the additional testing, the Neurotrack Cognitive Assessment Battery (N-CAB) was also administered. The neuropsychologist reviewed the other data provided by his staff and followed up with his own patient interviews, MRI scans, and blood work. The research protocol was approved for retrospective exemption by the institutional review board at the University of Arkansas for the analysis of de-identified data.

### Participants

All patients in this investigation were those coming to the clinic for their own health needs, and no one came independently to perform the N-CAB. The N-CAB is not a clinical examination requirement, and all patients were informed that they could opt in to the additional testing in order to gain further insights into their cognitive health. Cognitively, healthy participants and patients with diagnosed probable Alzheimer's disease were included in this study. Participants who had suspected Alzheimer's disease were evaluated by the study neuropsychologist and clinically diagnosed using the Diagnostic and Statistical Manual of Mental Disorders, 5th edition (DSM-5) diagnostic criteria for probable Alzheimer's disease (American Psychiatric Association, 2017). These participants will be referred to as individuals with probable Alzheimer's disease (pAD) because they received a clinical diagnosis, but this diagnosis was not confirmed by genetic testing or AD-specific *in vivo* biomarkers (e.g., CSF) (Jack et al., 2018). These patients' clinical diagnoses were determined through a combination of cognitive assessments and a clinical interview, including family history, conducted by the study neurologist. Cognitively, healthy participants were recruited from the family members and caregivers of clinic patients, as well as individuals who came to the clinic with concerns of cognitive decline but were subsequently deemed cognitively normal after a full evaluation from the study physician. If an individual was unable to give consent, as in the case of more severe cognitive impairment, their caregiver provided consent on their behalf. Given the RWE design of this retrospective analysis, no specific inclusion or exclusion criteria were applied to the participants in this study.

### Neurotrack cognitive assessment battery (N-CAB)

Before administration, the N-CAB was translated and localized to the Japanese language; a detailed description of this process can be found in the study by Glenn et al. (2019). Briefly, the initial translation was carried out by a bilingual (English and Japanese) speaker, native to the Japanese language and culture. Following this, the translation was reviewed by another bilingual individual, also native to the Japanese language and culture for a quality check of the translation. After the full translations were agreed upon, the Japanese version of all assessments was piloted with five individuals who did not speak English, only Japanese, to ensure a clear understanding of the assessments. Where necessary, adjustments were made based on the feedback of these individuals, and as a result, a final Japanese version of all assessments was produced. An Internet-enabled tablet was used to collect all study data and monitor the completion of all cognitive tasks. All cognitive tasks were able to be self-administered; however, clinic staff (nurses and medical assistants) were on hand to assist patients when necessary. As the assessment battery requires no training to administer and is scored objectively, it was not necessary for the clinic staff to be similar from patient to patient (i.e., there are no effects as a result of intra-individual training). The psychometric properties of the N-CAB have been evaluated in previous publications, and in-depth evaluations of the tasks can be found elsewhere (Bott et al., 2018; Gills et al., 2019, 2021; Myers et al., 2022); brief descriptions of each test are below.

#### Image pairs

Image Pairs is an eye-tracking task measuring visual recognition memory and learning and has shown good validity and reliability (*r* = 0.73, *p* < 0.001) (Bott et al., 2018; Gills et al., 2019, 2021). Briefly, Image Pairs tasks utilize a device-embedded camera for eye-tracking to assess visual recognition memory by quantifying the time a participant spends viewing novel images as opposed to previously viewed images; the eye-tracking scoring algorithm has previously been described in-depth (Bott et al., 2018; Gills et al., 2021). Participants are presented with Image Pairs and the task is broken into four phases. Phase 1 is the eye-tracking familiarization phase, consisting of 10 images. Phase 2 presents 10 pairs of images—one novel and one previously viewed in Phase 1. During this phase, participants are instructed to focus their gaze on the novel image (“Image Pairs (eye)”). Phase 3 is another familiarization phase where participants are presented with two images and are asked to remember the pair of images presented. Phase 4 consists of 25 pairs of images: 10 previously shown pairs from Phase 3, 10 mismatched pairs with images from Phase 3 (foil trials), and 5 pairs of novel images (sham trials). During this phase, participants are instructed to press “yes” or “no” buttons on-screen to identify whether the presented images were previously viewed together as a pair (“Image Pairs (hand)”). This test measures a participant's ability to learn and identify Image Pairs. The Phase 2 eye-tracking scores are reported as the percentage of time spent gazing at the novel image, and the Phase4 hand-response scores are reported as accuracy.

#### Symbol match

**Figure 1:**
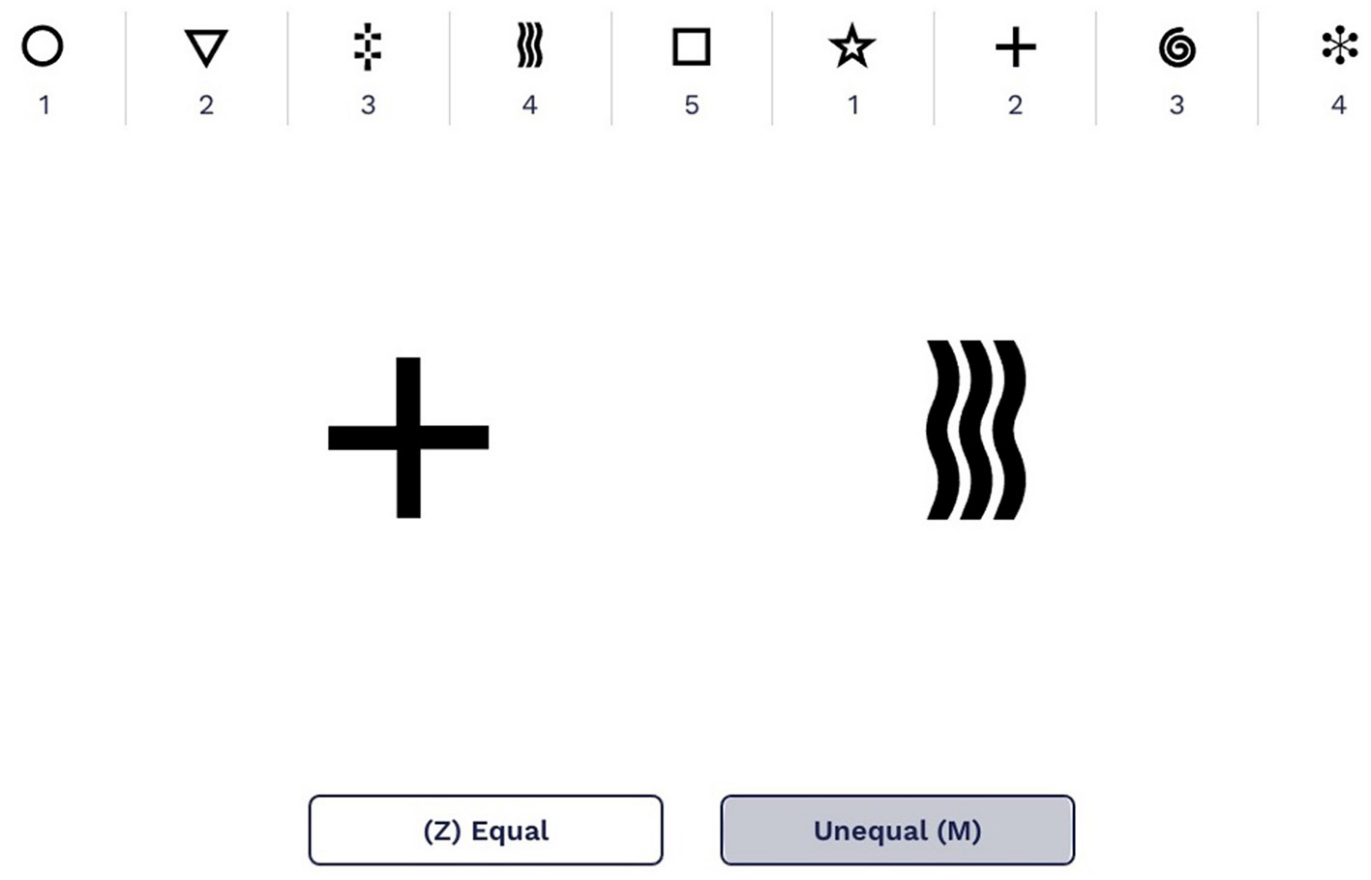
An example of the Symbol Match legend displaying the symbol/digit pairs with two larger symbols to compare. The screen shown here uses English numbers and words; in the study, all screens shown to participants were in Japanese.

Symbol Match is a validated 2-min assessment of processing speed and executive function (Kiely et al., 2014; Fellows and Schmitter-Edgecombe, 2020; Campitelli et al., 2023), with good test–retest reliability (*r* = 0.72, *p* < 0.001) (Myers et al., 2022). Participants are shown a legend with nine symbol/digit pairs; the first five pairs use numbers 1 through 5, and the last four pairs repeat numbers 1 to 5 (Figure 1). They are also shown a larger version of two symbols from the legend but different from each other in the middle of the screen and are instructed to determine whether two numbers associated with those symbols are equal or unequal. Participants are instructed to press the “Z” key if the numbers are of the same value or the “M” key if the numbers are of unequal value. Participants are allotted 2 min to complete as many trials as possible. Scores are reported as the number of correct trials minus the number of incorrect trials.

#### Path points

The Path Points test is used to assess executive function and has good test–retest reliability (ρ = 0.78, *p* < 0.001) (Myers et al., 2022). Similar to the traditional cognitive test, the Trail Making Test Part B (Lezak, 1995), Path Points is a digital version where participants connect a series of alternating numbers and letters from 1-A to 7-G (Smith, 1970); Japanese letters were used for this study. Scores are reported as the amount of time required to complete the 14 responses. Only correct responses are allowed.

### Traditional cognitive screening tests

#### Mini-mental state examination (MMSE)

The MMSE is a quick, clinician-administered evaluation that measures areas such as orientation to time and place, attention, concentration, short-term memory recall, language skills, visuospatial abilities, and visual and spatial relationships between objects (Dick et al., 1984). The MMSE demonstrates moderately high levels of reliability. It has been reported to be internally consistent. The MMSE has been found to have short-term test–retest reliability in patients with dementia, as well as long-term reliability in cognitively intact individuals. The MMSE has been shown to have construct validity, as it is moderately correlated with other dementia-screening examinations, as well as measures of general cognitive abilities (Hirsch, 2007; Bernard and Goldman, 2010). A total score out of 30 points is given, where a score of 23 or lower indicates cognitive impairment (Creavin et al., 2016).

#### Revised Hasegawa's Dementia scale (HDS-R)

The HDS-R consists of nine questions; question 1 is on age (1 point), question 2 on the date (4 points), question 3 on place (2 points), question 4 on the ability to repeat three familiar words (3 points), question 5 on two times of subtraction of 7 from 100 (2 points), question 6 on the backward repetition of three- and four-digit numbers (2 points), question 7 on the recall of the three words memorized in question 4 (6 points), question 8 on the immediate recall of five object pictures shown and hidden (5 points), and question 9 on the listing of 10 vegetable names (5 points). The full score on the HDS-R is 30 points. A score of 20 points or lower is considered to be an indicator of the presence of reduced function. To measure cognitive function accurately, both cooperation by the examinee and the skill of the examiner are necessary (Tsukamoto et al., 2009; Kounnavong et al., 2019).

### Outcome measures

Three specific outcomes were evaluated as part of this retrospective study.

#### Primary outcome

The primary outcome of this study was to evaluate the sensitivity and specificity of the individual tests and subsequently combined composites along with positive predictive value (PPV), negative predictive value (NPV), positive likelihood ratio (LR+), negative likelihood ratio (LR-), and area under curve (AUC) to correctly stratify healthy patients and patients with Alzheimer's disease.

#### Secondary outcome

The secondary outcome of this investigation was to investigate the relationship between the N-CAB to traditional cognitive tests, specifically the MMSE and HDS-R.

### Data analysis

Python version 3.9 and R version 4.1.3 were used to conduct all analyses. Descriptive statistics were calculated for age, sex, and education within the patient group, and Welch's t-test, the chi-squared test, and Fisher's exact test were used to measure between-group differences in these demographic variables (Table 1). The mean and standard deviation of the MMSE and HDS-R scores by the patient group are also provided. Due to many healthy subjects performing at the ceiling on the MMSE and HDS-R, the Wilcoxon rank-sum test was used to assess group differences in these two measures. Two subjects were much younger than the others (aged 37 and 43) and dropped from all analyses. Additionally, two N-CAB Image Pairs (eye) scores were removed for being much lower than chance (implying that the users have good enough memory to differentiate the familiar image). One pAD subject received a raw N-CAB Symbol Match score of −10, much lower than all other subjects. We deemed this score as not meaningfully reflecting any meaningful difference in performance from a raw score of 0 (both at the floor), and this score was rounded to a 0.

**Table 1:** Participant demographic data.

	**Healthy**	**pAD**	**Test statistic (*p* value)**
Total *N*	37	38	
Female sex: *N* (%)	19 (51.4)	23 (60.5)	0.32 (0.57)^a^
Age: mean (SD)	72.7 (7.5)	77.5 (6.6)	2.93 (< 0.001)^b^
Education: *N* (%)			0.43 (0.22)^c^
HS or less	15 (40.5)	22 (57.9)	
College or greater	8 (21.6)	5 (13.2)	
Unknown	14 (37.8)	11 (29.9)	
MMSE^d^	28.7 (1.5)	23.6 (2.3)	1,175.50 (< 0.001)^e^
HDS-R^d^	27.2 (2.2)	18.9 (2.7)	1,201.50 (< 0.001)^e^

a Chi-square test.

b Welch's t-test.

c Fisher's exact test between Healthy and AD groups with reported education.

d Mean with standard deviation in parentheses. 5 healthy subjects were missing MMSE and HDS-R scores.

e Wilcoxon rank-sum test.

#### Age-adjusted normative score calculation for individual scores

In the present sample, healthy subjects were significantly younger than those with pAD. Cognitive assessment scores, even among healthy subjects, may correlate negatively with age. If the N-CAB assessment scores are not age-adjusted, estimates of N-CAB assessment score differences by diagnosis and estimation of diagnostic accuracy performance of N-CAB assessments could be positively biased. Simple linear regression models were used to age-adjust the scores, fitting each N-CAB assessment separately. In these models, the raw scores for each assessment were regressed on age exclusively within the healthy subject group in the present sample.

Before fitting these linear regression models, histograms of raw score distributions for each N-CAB assessment were visually inspected. Two of these, the N-CAB Symbol Match and N-CAB Image Pairs (hand), appeared to have approximately normally distributed raw scores. The N-CAB Path Points raw scores were right-skewed and were transformed by taking their base 10 logarithms. The N-CAB Image Pairs (eye) raw scores were left-skewed. To identify an appropriate power transformation to make these scores more normal, they were Box-Cox transformed using different lambda values, and the log-likelihood was plotted for each. A lambda value of 4 was near the maximum-likelihood estimate; thus, the N-CAB Image Pairs (eye) raw scores were transformed by raising them to the power of 4. Post-transformation histograms of N-CAB Path Points and N-CAB Image Pairs (eye) scores appeared approximately normal.

After each of the N-CAB score + age models was fit, regression assumptions were checked. Residuals appeared homoscedastic in the residual versus fitted and scale-location plots. Additionally, the residuals were divided into two groups based on whether or not the age of the subject to which they belonged was above or below the median, and the equality of residual variance between the two age groups was evaluated via modified Levene's test. The results did not indicate unequal variance in residuals between age groups for any of the N-CAB score + age models (*p* > 0.05 for all tests). The Q-Q plots of residuals revealed them to be approximately normally distributed for each N-CAB assessment. The normality of the residuals for each N-CAB score + age model was also evaluated via Shapiro–Wilk test, and none of the results indicated non-normality of residuals (*p* > 0.05 for all tests).

Each healthy and pAD subject's age was input into each N-CAB score + age model for which they had an N-CAB score. The signed differences between the model-predicted scores, given the input age and actual scores, were taken as the un-normalized age-adjusted scores. Finally, for each model, the un-normalized age-adjusted scores were divided by the root mean squared error of the healthy subject's fitted scores to generate normalized age-adjusted scores for each subject for each N-CAB assessment. As a result, the healthy subject's normalized age-adjusted scores for each N-CAB assessment had a mean of approximately 0 and a standard deviation of ~1. The normalized age-adjusted N-CAB scores were used for all subsequent analyses.

Given the considerable proportion of missing responses (~38%) and the limited sample size of our healthy subject cohort, we decided against implementing such an adjustment. We also considered imputation methods for missing education but ultimately decided against them, again due to the high proportion of missing responses and limited sample of healthy subjects.

#### Composite score calculations

For each pair of N-CAB assessments and all four N-CAB assessments together, a simple composite score was calculated as the mean of the normalized age-adjusted scores. Due to real in-clinic administration, the full N-CAB battery was only administered time permitting, and as such, not all subjects received a score for each individual N-CAB assessment. If a subject was missing a member score for a particular composite, they did not receive that composite score. Table 3 contains the healthy and pAD sample sizes for each individual N-CAB assessment and composite.

#### Primary outcome

To evaluate differences in the N-CAB assessment scores by patient group for each individual N-CAB assessment, linear regression models were fit by regressing normalized age-adjusted scores on sex and patient group. Across all models, estimates of the dummy-coded sex coefficient were small, and *t*-test results did not indicate significant evidence that these estimates were non-zero. This, in combination with the lack of evidence of significant differences in sex proportion by patient group, led us to remove sex from the analysis of group differences. Accordingly, Welch's *t*-test was used to compare the group means in normalized age-adjusted scores between the healthy and pAD subjects. In addition, Hedge's *g* was calculated as a measure of effect size between groups. Cohen's conventions of *d* = 0.20 as small, *d* = 0.50 as medium, and *d* = 0.80 as large were used for the interpretation of the magnitude of group differences (Holm, 1979).

To evaluate the N-CAB assessments' capacity to distinguish between healthy and pAD subjects, receiver operating characteristic (ROC) analysis was performed. For each individual N-CAB assessment and composite of N-CAB assessments, for each score threshold, sensitivity, and specificity were calculated, and the optimal cutoff was defined to be the score threshold at which the Youden's J statistic (sensitivity + specificity – 1) was maximized. Sensitivity, specificity, accuracy, PPV, NPV, positive likelihood ratio (LR+), negative likelihood ratio (LR-), and ROC area under the curve (AUC) were calculated to evaluate the classification performance of each individual assessment or composite at its optimal cutoff score; 95% confidence intervals for sensitivity, specificity, and accuracy were calculated using the method described in Ref (Casella and Berger, 2021). Bootstrapping was used to calculate 95% confidence intervals for AUC, LR+, and LR-.

#### Secondary outcome

For comparison between the N-CAB and traditional assessments, correlations were calculated for each individual N-CAB/traditional assessment pair. Traditional assessments showed ceiling effects and could not be transformed to normal distributions; therefore, Spearman's correlations were used for all comparisons. *P*-values corresponding to these correlations were adjusted using the Holm correction for multiple comparisons (Holm, 1979). Cohen's conventions of *r* = 0.10 as small, *r* = 0.3 as medium, and r = 0.50 as large were used for the interpretation of the magnitude of correlation coefficients (Cohen, 2013).

In the interpretation of analyses for both the primary and secondary outcomes, we assumed estimates that were unbiased by missing N-CAB scores; for example, Assessment A having a higher sensitivity to pAD than Assessment B is not due to differences in which subjects have a score for Assessment A vs. which subjects have a score for Assessment B.

## Results

### Primary outcome

**Figure 2:**
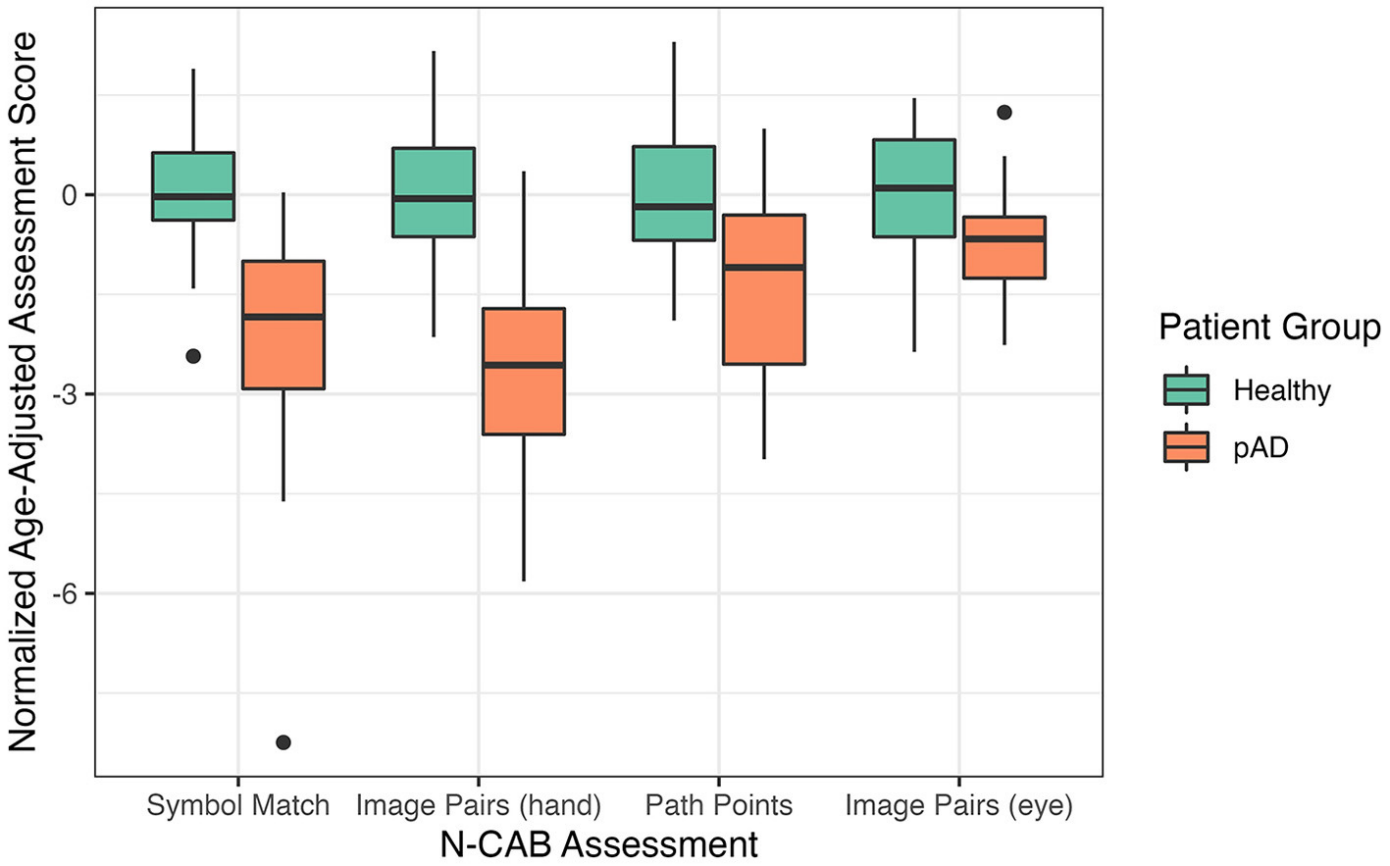
N-CAB scores by diagnosis.

Distributions of the normalized age-adjusted N-CAB assessment scores are shown in Figure 2. Welch's *t*-test results indicated pAD subjects performed worse than healthy subjects on all individual N-CAB assessments (Table 2). The largest group differences in scores were observed for Image Pairs (hand) with *g* = 2.17 and Symbol Match with *g* = 1.48, followed by Path Points with *g* = 1.12, and finally Image Pairs (eye) with *g* = 0.86. All effect size values were greater than the conventional “large” threshold of *d* = 0.8.

**Table 2:** Estimates of healthy vs. pAD N-CAB normalized age-adjusted score differences.

**N-CAB raw score descriptive statistics**				
	**Healthy**		**pAD**	
	**Mean (SD)**	**Min—max**	**Mean (SD)**	**Min—max**
Symbol match	21.48 (5.38)	7.00−30.00	10.68 (6.02)	0.00−21.00
Image pairs (hand)	0.70 (0.10)	0.52−0.92	0.43 (0.14)	0.12−0.72
Path points	29.55 (13.34)	9.94−70.59	64.31 (37.04)	18.26−139.73
Image pairs (eye)	0.76 (0.13)	0.31−0.91	0.65 (0.11)	0.45−0.86
	**Hedge's** ***g*** ^a^	***t***	***p*** **value**	
Symbol match	1.48 (0.86–2.11)	5.63	< 0.001	
Image pairs (hand)	2.17 (1.59–2.74)	9.49	< 0.001	
Path points	1.12 (0.44–1.60)	3.83	< 0.001	
Image pairs (eye)	0.86 (0.36–1.35)	3.56	< 0.001	

^a^Point estimate with 95% confidence interval in parentheses.

**Figure 3:**
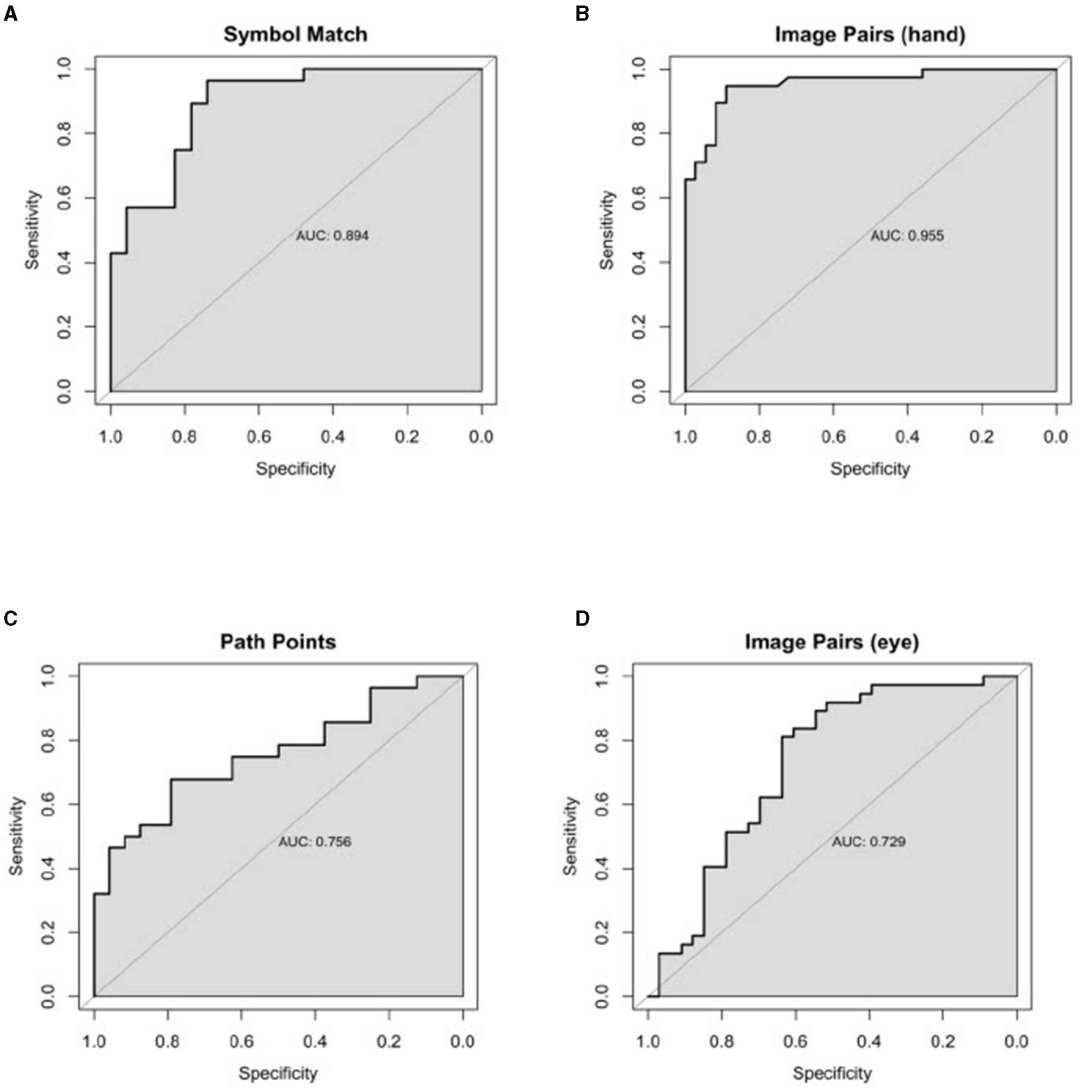
Individual Healthy vs. AD ROC curves for the following N-CAB assessment. **(A)** Symbol Match score; **(B)** Image Pairs Phase 4: the button press (hand) score; **(C)** Path Points score; and **(D)** Image Pairs Phase 2: the eye-tracking (eye) score.

**Figure 4:**
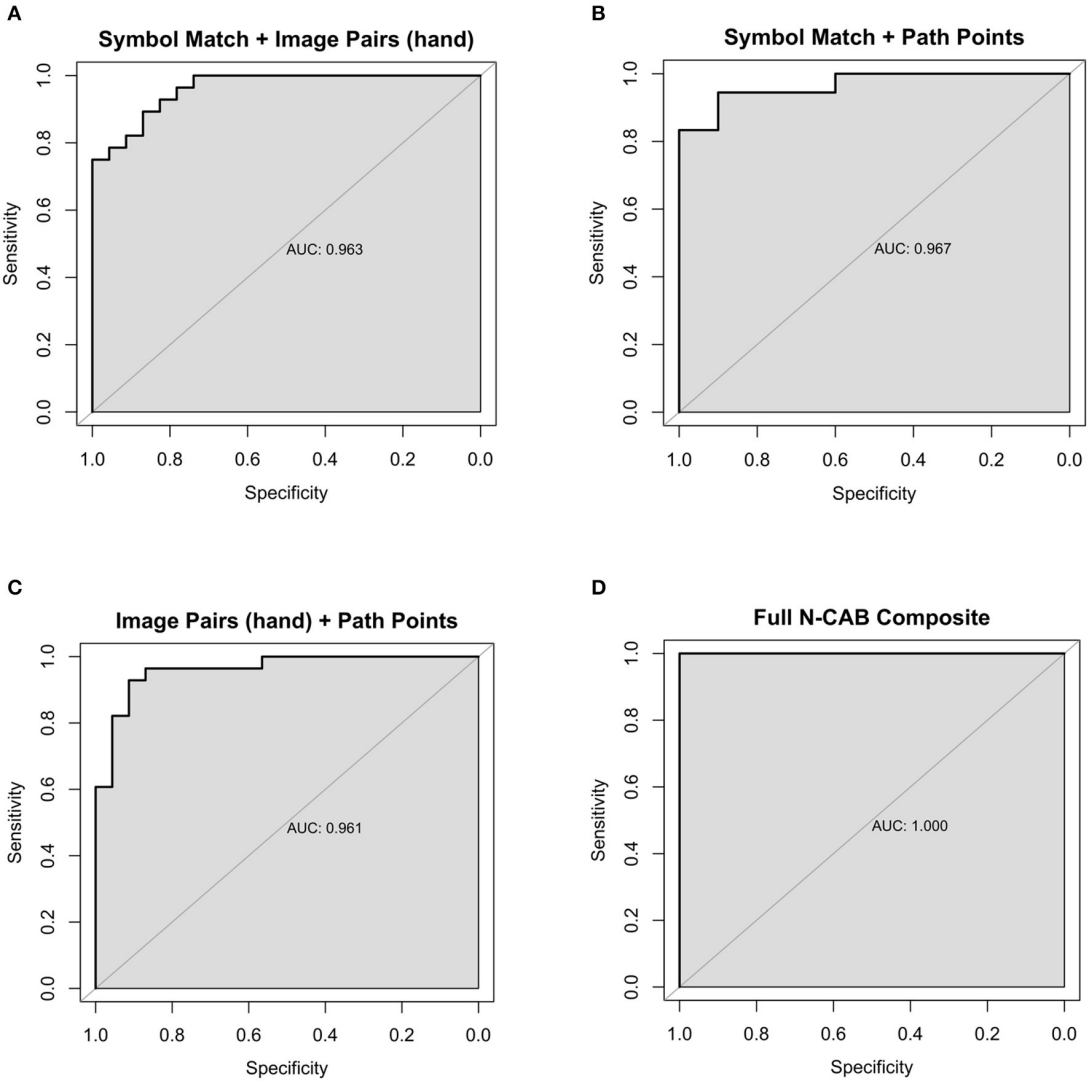
Composite Healthy vs AD ROC Curves for the following N-CAB assessments **(A)** Symbol Match & Image Pairs Phase 4 button press score (hand); **(B)** Symbol Match & Path Points; **(C)** Image Pairs (hand) & Path Points; **(D)** Full battery composite: Symbol Match, Path Points, Image Pairs (hand) and Image Pairs (eye).

Classification performance statistics for all N-CAB individual assessments and composites can be found in Table 3, and ROC curves can be found in Figures 3, 4, respectively. Individually, Image Pairs (hand) and Symbol Match had the strongest classification performance among individual N-CAB assessments at their optimal cutoffs: Image Pairs (hand) had a sensitivity and a specificity of 95% and 89%, respectively, and an AUC of 0.96. Symbol Match had a sensitivity and a specificity of 96% and 74%, respectively, and an AUC of 0.89. Path Points had lower sensitivity and specificity of 68% and 79%, respectively, and an AUC of 0.76. Among the individual assessments, Image Pairs (eye) performed the worst with a sensitivity and a specificity of 81% and 64%, respectively, and an AUC of 0.73.

**Table 3:** Classification performance of N-CAB assessments in detecting healthy vs. AD diagnosis.^a^

	**Healthy N**	**pAD N**	**Sens**	**Spec**	**PPV**	**NPV**	**Acc**	**LR+**	**LR-**	**AUC**	**Cutoff ^b^**
Symbol match	23	28	0.96 (0.82–1.00)	0.74 (0.52–0.88)	0.82 (0.66–0.92)	0.94 (0.74–1.00)	0.86 (0.74–0.94)	3.70 (2.16–10.62)	0.05 (0.00–0.17)	0.89 (0.80–0.97)	−0.27
Image pairs (hand)	36	38	0.95 (0.82–1.00)	0.89 (0.74–0.97)	0.90 (0.76–0.97)	0.94 (0.81–0.99)	0.92 (0.84–0.97)	8.53 (4.15–34.00)	0.06 (0.00–0.15)	0.96 (0.91–0.99)	−1.14
Path points	24	28	0.68 (0.48–0.83)	0.79 (0.58–0.92)	0.79 (0.58–0.92)	0.68 (0.48–0.83)	0.73 (0.59–0.84)	3.26 (1.62–11.88)	0.41 (0.19–0.68)	0.76 (0.62–0.88)	−0.79
Image pairs (eye)	33	37	0.81 (0.65–0.91)	0.64 (0.45–0.80)	0.71 (0.56–0.84)	0.75 (0.56–0.88)	0.73 (0.61–0.82)	2.23 (1.47–4.14)	0.30 (0.11–0.55)	0.73 (0.60–0.85)	−0.29
Symbol match + image pairs (hand)	23	28	0.89 (0.72–0.98)	0.87 (0.68–0.97)	0.89 (0.72–0.98)	0.87 (0.68–0.97)	0.88 (0.77–0.95)	6.85 (3.07–23.00)	0.12 (0.00–0.28)	0.96 (0.91–0.99)	−1.02
Symbol match + path points	10	18	0.94 (0.74–1.00)	0.90 (0.56–1.00)	0.94 (0.74–1.00)	0.90 (0.56–1.00)	0.93 (0.78–0.99)	9.44 (2.69–13.85)	0.06 (0.00–0.21)	0.97 (0.89–1.00)	−0.37
Symbol match + image pairs (eye)	22	28	0.79 (0.59–0.91)	0.86 (0.66–0.97)	0.88 (0.69–0.97)	0.76 (0.56–0.89)	0.82 (0.69–0.91)	5.76 (2.53–19.00)	0.25 (0.09–0.45)	0.88 (0.79–0.96)	−0.79
Image pairs (hand) + path points	23	28	0.93 (0.78–0.99)	0.91 (0.72–0.99)	0.93 (0.78–0.99)	0.91 (0.72–0.99)	0.92 (0.82–0.98)	10.68 (4.11–25.87)	0.08 (0.00–0.20)	0.96 (0.91–1.00)	−0.69
Image pairs (hand) + image pairs (eye)	33	37	0.84 (0.68–0.93)	0.88 (0.72–0.96)	0.89 (0.74–0.97)	0.83 (0.67–0.93)	0.86 (0.76–0.93)	6.91 (3.36–27.39)	0.18 (0.06–0.34)	0.93 (0.86–0.98)	−0.96
Path points + image pairs (eye)	21	27	0.67 (0.46–0.82)	0.95 (0.77–1.00)	0.95 (0.74–1.00)	0.69 (0.50–0.84)	0.79 (0.65–0.90)	14.00 (3.68–19.57)	0.35 (0.17–0.55)	0.87 (0.76–0.95)	−0.67
Full N–CAB composite	10	18	1.00 (0.82–1.00)	1.00 (0.72–1.00)	1.00 (0.82–1.00)	1.00 (0.72–1.00)	1.00 (0.88–1.00)	undef ^c^	0.00 (N/A) ^c^	1.00 (N/A) ^c^	−0.38

a Point estimate with 95% confidence interval in parentheses.

b Optimal score cutoff, at which Youden's J is maximized.

c There was perfect separation between cases and non cases. The denominator in the LR+ calculation is 1 - specificity, so LR+ is undefined. Bootstrapping LR- results in both upper and lower CI being 0. Bootstrapping procedure yields AUC of 1 across all samples with replacement and is invalid.

When evaluating assessment pairs, Symbol Match + Image Pairs (hand) and Path Points + Image Pairs (hand) composites performed similarly to Image Pairs (hand) individually. Image Pairs (hand) + Image Pairs (eye) performed marginally worse than Image Pairs (hand) individually but better than Image Pairs (eye) individually. Symbol Match + Path Points (sensitivity = 94%, specificity = 90%, AUC= 0.97) performed better than each assessment individually and had near-perfect classification performance; however, the particularly small number of subjects for the Symbol Match + Path Points composite should be noted here. Symbol Match + Image Pairs (eye-tracking) (sensitivity = 79%, specificity = 86%, AUC = 0.88) performed marginally worse than Symbol Match individually. Path Points + Image Pairs (hand) (sensitivity = 93%, specificity = 91%, AUC= 0.96) showed the largest improvement in classification performance over its individual components, which had the most room for improvement. The full N-CAB composite of all four outcome metrics had perfect classification performance (sensitivity = 100%, specificity = 100%, AUC= 1.00) but with the same considerably smaller sample size as the Path Points + Symbol Match composite.

### Secondary outcome

Holm-corrected *p*-values from Spearman correlations between traditional cognitive assessments (MMSE and HDS-R), and all N-CAB assessments were all significant at α < 0.01. The strongest correlations were between the N-CAB Symbol Match and the HDS-R (r = 0.77), MMSE (r = 0.72), and Image Pairs (hand) with HDS-R (r = 0.73) (Table 4).

**Table 4:** N-CAB vs. traditional assessment correlations.^ab^

	**Symbol match**	**Image pairs (hand)**	**Path points**	**Image pairs (eye)**
MMSE	0.72 (0.56–0.83)^***^	0.70 (0.55–0.80)^***^	0.44 (0.18–0.65)^**^	0.49 (0.28–0.65)^***^
HDS-R	0.77 (0.62–0.86)^***^	0.73 (0.59–0.82)^***^	0.43 (0.17–0.64)^**^	0.38 (0.15–0.57)^**^

a Spearman's correlation coefficient. Holm adjusted ^**^P < 0.01; ^***^P < 0.001.

b Point estimate of correlation coefficient with 95% confidence interval in parentheses.

## Discussion

The primary aim of this study was to (1) evaluate diagnostic accuracy by investigating the sensitivity and specificity of the individual tests, composite scores, and positive likelihood ratio of the battery to correctly evaluate individuals with cognitive decline and (2) investigate the relationship between the N-CAB and traditional cognitive tests. This was completed by incorporating the N-CAB into a standard clinical assessment performed within a Japanese-based neuropsychology clinic. Overall, pAD patients performed worse than healthy individuals on all N-CAB assessments. Our results demonstrate that the Image Pairs hand-response phase (Phase 4) has the highest diagnostic accuracy, closely followed by the Symbol Match assessment. Additionally, all N-CAB assessments had moderate-to-strong and significant correlations with the traditional cognitive tests.

The N-CAB individual assessments demonstrated the ability to distinguish between cognitively healthy patients and patients with diagnosed Alzheimer's disease with a high degree of accuracy. Specifically, the hand-response phase of Image Pairs was able to distinguish between healthy and Alzheimer's patients with 95% sensitivity and 89% specificity, and the Symbol Match assessment had 96% sensitivity and 74% specificity, suggesting that, on an individual level, they are acceptable to use in a screening and Image Pairs (hand) + Path Points; however, the former has a low sample size and should be interpreted as preliminary results. When all four N-CAB assessments [Symbol Match, Path Points, Image Pairs (hand), and Image Pairs (eye)] were considered for the full N-CAB composite score, the sensitivity and specificity to AD were 100%.

Recent research has suggested that the optimal cutoff for sensitivity and specificity is >80% in the population with dementia when evaluating the diagnostic accuracy of a dementia screening tool (Hoops et al., 2009). Considering these criteria, Image Pairs (hand) had the best diagnostic accuracy overall as an individual assessment tool, suggesting that this task alone may be an acceptable screening tool for dementia. The Symbol Match assessment also approached this optimal diagnostic accuracy. Together, these two assessments have acceptable diagnostic accuracy with a sensitivity of 89% and a specificity of 87%; all other diagnostic accuracy metrics are above the optimal level of 80%, suggesting that these two assessments used in combination may be an optimal screening tool for dementia. Additionally, the Symbol Match and Path Points pairing resulted in a sensitivity of 94%, an LR- of 0.06, a specificity of 90%, and an LR+ of 9.44, further suggesting that the addition of the N-CAB Path Points to a dementia screening test battery would be beneficial in the earlier detection of dementia.

Both Image Pairs (hand) and Symbol Match demonstrated significantly strong and positive correlations with the two traditional clinical cognitive assessments used in this study. This demonstrates that, as scores increase on the traditional cognitive assessments, representing better cognitive performance, the scores on Symbol Match and Image Pairs (hand) also increase, representing better performance on these digital cognitive assessments. The MMSE is a widely used screening tool in primary care clinics with variable sensitivity (66-97%) and specificity (70–100%) levels being reported for dementia (Nasreddine et al., 2005; Mitchell, 2008; O'Bryant et al., 2008; Creavin et al., 2016); however, a 2009 meta-analysis demonstrated that the MMSE has low diagnostic accuracy in primary care settings where dementia prevalence is relatively low, as well as in specialist memory clinic settings where dementia prevalence is high. The MMSE is also known to have limitations due to its age and education bias, as well as a cultural and socioeconomic background bias (Carnero-Pardo, 2013). The HDS-R reports a cutoff score of 20/21 out of a total 30 points, with a sensitivity of 90% and a specificity of 82% for the detection of dementia [Dick et al., 1984; The Revised Hasegawa's Dementia Scale (HDS-R), 1994]; additionally, the HDS-R has a high correlation with the MMSE and slightly higher sensitivity but lower sensitivity than the MMSE; however, literature for both traditional cognitive assessments have reported that some cognitive impaired patients can score above the cutoff scores, and some healthy individuals can score below the cutoff scores, suggesting that these traditional methods may not be the best option for screening patients for dementia (Dick et al., 1984; The Revised Hasegawa's Dementia Scale (HDS-R), 1994). The N-CAB full composite battery provides better diagnostic accuracy than the MMSE and HDS-R in a similar amount of administration time. Given the limitations of the traditional cognitive assessments of dementia, and in conjunction with the diagnostic accuracy results in this study, the N-CAB Symbol Match, Path Points, and Image Pairs (hand) are appropriate alternatives for in-clinic dementia screening.

There are a few limitations associated with this study. First, not all participants completed the N-CAB and others only completed a subset of the tests, which resulted in a limited sample size. This was expected and based on the time in the clinic that was available for the assessment protocol and the patient's desire to continue the battery, which is expected given the RWE nature of this study; however, the small sample size should be recognized as a limitation of this study. Second, these data were collected from native Japanese individuals and it cannot be guaranteed that all results would directly extrapolate to other populations; however, given the limited language included in the tests and the fact that only culturally agnostic symbols and/or numbers were used in the N-CAB, a reasonable assumption can be made as to cultural/ethnic crossover. Finally, there is the potential for selection bias as the individuals going to a neuropsychology clinic are naturally worried about their cognitive health. However, that also implies that even the healthy individuals were experiencing some level of subjective cognitive decline to even go to the clinic, thus making the comparisons between healthy and impaired individuals all the more interesting.

In conclusion, all N-CAB assessments (Image Pairs, Symbol Match, and Path Points) have good individual diagnostic accuracy; however, the combination of all N-CAB assessments' high diagnostic accuracy, alongside the strong and significant correlation between the N-CAB assessments and the traditional cognitive assessments. The N-CAB Image Pairs, Symbol Match, and Path Points assessments may provide a more cost and time-effective alternative dementia screening method for the elderly population.

## Data availability statement

The raw data supporting the conclusions of this article will be made available by the authors, without undue reservation.

## Ethics statement

The studies involving humans were approved by the Institutional Review Board at the University of Arkansas. The studies were conducted in accordance with the local legislation and institutional requirements. Written informed consent for participation was not required from the participants or the participants' legal guardians/next of kin in accordance with the national legislation and institutional requirements.

## Author contributions

JG, KB, JMy, JA, and JMc assisted in the drafting of the manuscript. KO assisted with data collection. SO assisted with patient recruitment and data collection. All authors contributed to the article and approved the submitted version.
